# Patient Perception of Skin Cancer Reduction by Nicotinamide Correlates With Use

**DOI:** 10.7759/cureus.44403

**Published:** 2023-08-30

**Authors:** Justine G Schneider, Mandy Majidian, Ronald L Moy

**Affiliations:** 1 Dermatology, The Ohio State University College of Medicine, Columbus, USA; 2 Dermatology, Tulane University School of Medicine, New Orleans, USA; 3 Dermatology, Moy-Fincher-Chipps Facial Plastics and Dermatology, Beverly Hills, USA

**Keywords:** skin cancer, melanoma, squamous cell carcinoma, basal cell carcinoma, vitamin b 3, nicotinamide, skin cancer prevention, nonmelanoma skin cancer

## Abstract

Introduction

Nicotinamide (Vitamin B3) has been shown to reduce the rate of non-melanoma skin cancers by 23%, yet most patients do not know that this supplement reduces skin cancer. Understanding patient beliefs about skin cancer reduction attributed to nicotinamide is important to appropriately counsel patients on oral supplement use and ultimately to prevent non-melanoma skin cancers.

Objective

The objective of this study was to determine the association between nicotinamide use and perceived efficacy in skin cancer reduction.

Methods

Patients who underwent Mohs surgery in 2019 were sent an online survey assessing nicotinamide use, efficacy compared to sunscreen, and perceived skin cancer risk reduction.

Results

Data from 50 surveys revealed a perceived risk reduction attributed to nicotinamide of 31.2% for basal cell carcinoma (BCC), 30.2% for squamous cell carcinoma (SCC), and 24.3% for melanoma. In the subset of respondents taking nicotinamide, the perceived risk reduction was significantly higher at 41.2% for BCC and 38.3% for SCC (p<0.05) and positively correlated with reported nicotinamide use (p<0.05). The perceived risk reduction of melanoma was not significantly increased in patients taking nicotinamide (31.6%); however, the perceived risk reduction was correlated with nicotinamide use (p<0.05). In addition, 15.6% of respondents believed that nicotinamide was more effective than sunscreen at preventing skin cancer.

Conclusion

A larger perceived reduction of non-melanoma skin cancers attributed to nicotinamide is associated with increased oral nicotinamide use. Better patient education regarding the reduction of skin cancers with oral nicotinamide will need to be implemented to change patients’ perceptions of the value of nicotinamide.

## Introduction

The major underlying cause of skin cancer is ultraviolet light-induced DNA damage secondary to excessive sun exposure. Current recommendations for skin cancer prevention include the use of sunscreen with a minimum SPF of 30 [1,2]. However, for those individuals who have already accumulated significant sun damage, interventions other than sunscreen must be taken into consideration in order to reverse past damage and prevent skin cancer.

When evaluating sunlight-induced skin damage, the ultraviolet (UV) component has the most deleterious effects on DNA. UV radiation induces mutations in various genes that protect the skin while also inducing pyrimidine dimer lesions in cellular DNA. Once this DNA damage has occurred, repair mechanisms must be initiated prior to replication in order to prevent the development of skin cancers, primarily basal cell carcinoma (BCC) and squamous cell carcinoma (SCC) [3].

Nicotinamide (Vitamin B3) is a water-soluble amide form of niacin (nicotinic acid) and can be obtained in the diet from foods such as fish, poultry, eggs, and grains [4]. While niacin and nicotinamide are related, they should not be viewed as synonymous. Nicotinamide enhances the rate of DNA repair, prevents UV-induced immunosuppression, and, in a seminal study, was shown to reduce the rate of non-melanoma skin cancers by 23% in a phase three, double-blind, randomized controlled trial published in the New England Journal of Medicine in 2015 [5-7]. By providing the precursor molecules for key components to produce adenosine triphosphate (ATP), nicotinamide also enhances DNA repair by replenishing cellular energy after UV damage [6,7]. Current research also suggests that nicotinamide plays a role in blocking inflammatory cell activation and influences cell life span, survival, and cancer progression [8,9]. Studies have reported that patients with a history of non-melanoma skin cancer often present with vitamin deficiencies; thus, it has been proposed that vitamin replacement can be of great importance in these patients [10,11]. The recommended dose of oral supplementation with nicotinamide is 500 mg twice daily [5].

Although data supporting nicotinamide for skin cancer prevention exists, understanding patient beliefs about skin cancer reduction attributed to nicotinamide is very important in order to appropriately counsel patients on supplement use and decrease the incidence of skin cancer. Patient perception regarding the efficacy of nicotinamide has not been evaluated to date, and it is not known if patient perception correlates with use. We hypothesize that a larger perceived reduction of non-melanoma skin cancer attributed to nicotinamide would be associated with increased oral nicotinamide use.

## Materials and methods

Patient selection

A retrospective review of the electronic medical record database at a dermatology private practice was conducted to identify all patients who underwent Mohs micrographic surgery at this practice in 2019. The study was conducted at Moy-Fincher-Chipps Facial Plastics & Dermatology located in Los Angeles, California. Patients were selected if they underwent Mohs surgery for BCC, SCC, and/or melanoma; patients who underwent Mohs surgery for other diagnoses were excluded. Participants were required to be at least 18 years of age.

Survey design

An online survey was designed to gather patient perceptions of nicotinamide. Basic demographics were obtained, including age, gender, and previous skin cancer history (BCC, SCC, and/or melanoma). The survey queried knowledge regarding skin cancers (most/least common, highest/lowest mortality), current nicotinamide use, efficacy attributed to nicotinamide, and effectiveness relative to sunscreen. For these questions, responses were multiple-choice. The perceived risk reduction of nicotinamide for BCC, SCC, and melanoma was obtained from each participant via patient-reported percentages. Specifically, participants were asked how much they believed nicotinamide reduced the risk of each type of skin cancer (BCC, SCC, and melanoma) and then were asked to respond with a percentage ranging from 0% to 100%. Patient comfort with percentages was determined via the Subjective Numeracy Scale (SNS), therefore upholding self-reported numerical responses [12,13]. The Subjective Numeracy Scale is an eight-question scale that does not require mathematics and has no incorrect or correct answers. Instead, the first section is comprised of four questions assessing participants’ numerical ability in various contexts, while the second section is comprised of four questions assessing participants’ preference for the presentation of numerical information [12,13]. The survey on nicotinamide perceptions was determined to be exempt from institutional review board (IRB) oversight by Advarra IRB.

Survey distribution and data collection

The survey was conducted in 2019. Email invitations to complete the survey were sent to eligible patients. A skin care product valued at less than $50 was offered as an incentive for the completion of the survey. Study responses were collected anonymously using a secure, web-based software platform designed to support data capture.

Data analysis

Results were tabulated in Excel and subsequently analyzed. The student's two-tailed T-test was used to determine statistical significance between groups. Pearson’s test was used to determine correlation. Results were considered statistically significant at a p-value < 0.05.

## Results

Participants

Online surveys were sent to prior Mohs micrographic surgery patients. The response rate was 59.1%; survey responses from 50 patients were received and analyzed. Participants had an average age of 66 years (range 46-92) and were 60% female. All participants had a previous history of skin cancer that required Mohs surgery: 76% of patients had a prior BCC, 48% SCC, and 10% melanoma (Table 1). Results showed that 64% of participants believed that an individual had the highest risk of developing BCC, while 66% believed developing melanoma carried the lowest risk. With respect to mortality, 90% of participants believed melanoma had the highest mortality, while 66% of respondents believed that BCC had the lowest mortality.

**Table 1:** Patient skin cancer history and nicotinamide use Types of skin cancer are present in all patients, including those who take nicotinamide and those who do not. *Percent calculated from 21 nicotinamide (nic) users and 26 nicotinamide non-users. Two BCC and one melanoma did not respond to nicotinamide use question. BCC: basal cell carcinoma; SCC: squamous cell carcinoma

	BCC	SCC	Melanoma
Overall personal history, n (% total)	38 (76%)	24 (48%)	5 (10%)
+ nicotinamide personal history, n (% nic users)*	17 (81%)	11 (52%)	2 (10%)
- nicotinamide personal history, n (% nic non-users)*	19 (73%)	13 (50%)	2 (8%)

Nicotinamide use

Forty-five percent of participants (n=21) who responded to the nicotinamide use question (no response from two patients with BCC and one with melanoma) acknowledged current oral nicotinamide use, while 55% reported no current use. Of the 21 participants who used nicotinamide, 60% were female, 10% had a prior melanoma, 52% had a prior SCC, and 81% had a prior BCC (Table 1). There was no significant difference in the likelihood of nicotinamide use among patients with a history of multiple types of skin cancer (for example, BCC and SCC) compared to only one type of skin cancer (for example, only SCC) (p=0.69). On average, each skin cancer group was close to evenly split between nicotinamide users and non-users. Age and sex were similar between nicotinamide users and non-users (p=0.76 and p=0.23, respectively). 

Nicotinamide and skin cancer prevention

All study participants were asked to provide an estimate (percent) of nicotinamide's efficacy in reducing the risk of skin cancer. Analysis of survey data revealed an overall perceived risk reduction attributed to nicotinamide of 31.2% for BCC, 30.2% for SCC, and 24.3% for melanoma (Table 2). In respondents currently taking nicotinamide, the perceived risk reduction was significantly higher at 41.2% for BCC and 38.3% for SCC versus each respective overall group (p<0.05) and positively correlated with reported nicotinamide use (p<0.05). For nicotinamide non-users, the perceived ability of nicotinamide to reduce the risk of BCC and SCC was significantly lower than the respective overall groups at 23.0% and 25.4% (p<0.05). The perceived risk reduction of melanoma in those taking nicotinamide (31.6%) was not significantly increased compared to the overall group (p=0.09); however, perceived risk reduction did correlate with nicotinamide use (p<0.05). In addition, 15.6% of respondents believed that nicotinamide was more effective than sunscreen at preventing skin cancer.

**Table 2:** Perceived risk reduction of nicotinamide for skin cancer Perceived risk reduction in all patients, those who take nicotinamide and those who do not. Percentages listed are means. *Significant at p<0.05 BCC: basal cell carcinoma; SCC: squamous cell carcinoma

	BCC	SCC	Melanoma
Overall perceived risk reduction, %	31.2%	30.2%	24.3%
+ nicotinamide, % risk reduction (p-value)	41.2% (0.033)*	38.3% (0.046)*	31.6% (0.09)
- nicotinamide, % risk reduction (p -value)	23.0% (0.026)*	25.4% (0.046)*	15.6% (0.059)
Correlation, R score (p-value)	0.44 (0.006)*	0.39 (0.015)*	0.35 (0.041)*

## Discussion

This study sought to evaluate patient perceptions of skin cancers, nicotinamide use, the efficacy of nicotinamide in the prevention of skin cancers, and the comparison of nicotinamide to sunscreen in terms of prevention. The initial hypothesis was confirmed by our results, which demonstrate that a greater perceived reduction of non-melanoma skin cancers attributed to nicotinamide is associated with increased oral nicotinamide use. However, 84.4% of patients believed that nicotinamide was less effective than sunscreen at preventing skin cancer, suggesting that patients are not properly educated on alternative cancer prevention methods. This is important because, although sunscreen prevents DNA damage from initially occurring by reducing exposure, nicotinamide helps repair existing DNA damage by enhancing two different DNA repair pathways for UV-induced mutations [14].

A handful of studies have examined physician perceptions of systemic/oral photoprotection, including nicotinamide [15-17]. In surveys evaluating nicotinamide prescribing patterns among dermatologists, the percentage of respondents who recommend nicotinamide for skin cancer prevention ranged between 76.9% and 84.2% [15,16]. A 2021 survey of 1,500 Mohs surgeons examined the relationship between nicotinamide prescribing habits and physician characteristics [16]. Twenty percent of respondents recommended nicotinamide to greater than 100 patients in the previous year; 63.8% of respondents reported no concern over nicotinamide’s long-term safety profile. Physician characteristics associated with not recommending nicotinamide to patients for cancer prevention included being in practice for more than 10 years or having safety concerns over long-term nicotinamide use. Arzeno et al. similarly found that dermatologists in practice for up to five years were 7.41 times more likely to recommend nicotinamide compared to those in practice for 15 years or longer (p=0.017) [15]. Furthermore, dermatologists who were aware of the seminal 2015 New England Journal of Medicine study were 11.2 times more likely to recommend nicotinamide compared to those unaware of the study (p=0.0007) [15]. Of note, the authors did not find a statistically significant association between practice setting (private practice, academic institution, or other) and the likelihood of recommending nicotinamide. The results of these studies are critical because patient perceptions of nicotinamide’s efficacy and safety are likely influenced by physician perceptions and recommendations. Thus, gathering more evidence supporting the long-term safety of nicotinamide and informing physicians may increase patients’ perceived effectiveness of nicotinamide.

To our knowledge, the current study is the first to evaluate patient perceptions of nicotinamide. Multiple studies exist regarding patient beliefs about sunscreen efficacy for skin cancer prevention [18-22]. A survey of patients with a history of skin cancer reported that greater perceived skin cancer risk reduction was associated with increased sunscreen use. Similarly, our current study reports a positive correlation between perceived skin cancer risk reduction and nicotinamide use. Sharma et al. reported that participants believed sunscreen would decrease their risk of developing basal cell carcinoma by 61.1%, squamous cell carcinoma by 59.4%, and melanoma by 59.5% [22]. Interestingly, the perceived risk reduction of sunscreen is approximately double the perceived risk reduction of nicotinamide found in our study (BCC 31.2%, SCC 30.2%, melanoma 24.3%). Potential reasons for this include the novelty and general lack of familiarity with nicotinamide [23]. The first key study documenting nicotinamide for skin cancer prevention was published in 2015, and research on its efficacy, cost-effectiveness, and safety is ongoing. In contrast, sunscreen has been well-known by the public for decades to decrease the risk of skin cancer [2,24-27]. Additionally, widespread communication and education on the importance of sunscreen for skin cancer prevention occur through many avenues, such as doctors, family, friends, news, advertisements, and social media. In a study on patterns of skin cancer communication among 600 primary care patients, 66% reported discussing skin cancer risks with their families [18]. Information about skin cancer was most commonly communicated by doctors (54%), followed by friends (49%), partners/spouses (43%), and other family members (38%). The most commonly discussed topic related to skin cancer was the use of sun protection. Our study demonstrates that while education about nicotinamide provided by physicians is important, discussions among friends and family will also be instrumental in spreading nicotinamide awareness.

One limitation of this study was the relatively low response rate to the online survey. This could be due to the older patient population, who may be less adept at navigating a website-based survey. Another factor to consider is that patients at this private dermatology practice are often educated on nicotinamide and encouraged to incorporate the supplement into their routines for skin cancer chemoprevention. While this could be considered a potential source of bias, it also further supports the concept that proper patient counseling increases patient confidence in the efficacy of the product and thus increases patient compliance. It also supports the notion that education and counseling on skin cancer prevention should focus on multiple approaches, incorporating both sunscreen and additional methods such as nicotinamide that repair previously damaged DNA.

## Conclusions

Overall, this study is significant because it illustrates the relationship between taking a supplement and believing in its efficacy. Specifically, our results should encourage providers to educate patients and their families on nicotinamide and its link to skin cancer prevention. Future studies could investigate the perceived efficacy of nicotinamide in a population that has no history of skin cancer and has no prior knowledge of nicotinamide. Additionally, evaluating whether patients understand the repair of past DNA damage with nicotinamide compared to the prevention of future damage with sunscreen would help to further elucidate the cause of patients’ perception of nicotinamide.
